# Study on Clinicopathological Features and Risk Factors of Patients with Multiple Primary Breast Cancers and Thyroid Disease

**DOI:** 10.1155/2023/3133554

**Published:** 2023-04-26

**Authors:** Jie Li, Yonghong Liu, Jian Jin, Qingfeng Shi, Yanting Ji, Bo Zhang, Pengfei Hu

**Affiliations:** ^1^Cangzhou Central Hospital, Cangzhou 061000, China; ^2^Hebei Medical University, Shijiazhuang 050017, China

## Abstract

**Objective:**

To explore the clinicopathological features and risk factors of patients with multiple primary breast cancers and thyroid disease.

**Method:**

An analytic approach of the reviewing method was adopted to analyze the clinical data of 80 breast cancer patients who were admitted to our hospital from January 2020 to January 2022. They were divided into an observation group (breast cancer with thyroid lesions) and a control group (simple breast cancer) according to whether the clinical data were accompanied with thyroid lesions to compare the clinical characteristics, pathological types, staging characteristics, and molecular biological characteristics of the two groups and to research the risk factors of the two groups.

**Result:**

(1) In the comparison of clinical data, the number of people aged ≥60 in the observation group was higher than that in the control group, and there was significant difference between the groups in the menopausal status data (*P* < 0.05). There was no statistical difference between the observation group and the control group in the comparison of clinical data of the body mass index, pregnancy frequency, labor frequency, and abortion history (*P* > 0.05). (2) In the comparison of pathological type and staging data, there was no statistical difference in the comparison of data on the pathological type, histological grade, T staging, N staging, and TNM staging between the observation group and the control group (*P* > 0.05). (3) In the comparison of data on molecular biology characteristics, there was a statistical difference in the nuclear proliferation antigen data between the observation group and the control group (*P* < 0.05). There was no statistical difference in the comparison of data on the estrogen receptor, progesterone receptor, human epidermal growth factor receptor-2, and molecular typing between the observation group and the control group (*P* > 0.05). (4) Logistic regression analysis showed that age, menopausal status, and nuclear proliferation antigen index were the high-risk inflammatory factors for combined thyroid lesions (*P* < 0.05).

**Conclusion:**

For patients with simple breast cancer, age, menopausal status, and nuclear proliferation antigen index are risk factors for combined thyroid lesions. Therefore, clinical attention should be paid to the above factors in the process of clinical treatment, and early-risk screening should be performed to achieve the purpose of improving the prognosis to the greatest extent.

## 1. Introduction

With the continuous development of social lifestyle, more and more factors will affect people's physical and mental health [1, 2]. Among them, the most obvious factors are biological genetic factors, environment, and lifestyle, which have also led to the increasing incidence and mortality of cancer in recent years. However, the form of cancer prevention and treatment all over the word is not very ideal. According to some research reports [3, 4], the tumor burden in China keeps increasing, and the number of deaths from cancer exceeds 2.3 million throughout the year. Just from the perspective of breast cancer, the number of new breast cancer cases in China is 280,000 in recent years, and the patients are mainly aged 45–55 years old. Compared with other European and American countries, the onset time in China is about ten years ahead of schedule. Therefore, breast cancer has become one of the most common cancer types among women in China, and it is also an important cause of death for women in our country, and the incidence has been rising every year. However, there is no unified specific cause of breast cancer, and most of the medical researchers believe that it is related to heredity, age, body mass index, and living habits [5, 6]. Nowadays, the levels of medical diagnosis and treatment have been developed in China, but breast cancer still seriously affects the physical and mental health of female residents, so it is particularly important to improve the diagnosis and treatment technology for breast cancer and its related complications. Thyroid diseases mainly include hyperthyroidism, hypothyroidism, thyroiditis, and thyroid tumor, which often occur in the female population. Among them, the thyroid nodular lesion is a very common hyperplastic disease clinically. Data [7] shows that the incidence of thyroid disease is closely related to gender, age, heredity, iodine intake, etc. Both the thyroid and breast belong to hormone-responsive organs, and they are regulated by the hypothalamic and pituitary systems, so it can be deduced that the endocrine function changes of the body are closely related to the occurrence and development of these two diseases. Estrogen and thyroid hormones can lead to interaction and influence on breast cancer and thyroid disease. For example, estrogen can affect physiological and pathological changes of the thyroid, thus leading to thyroid disease. The thyroid hormone, in turn, can also increase the risk of breast cancer. Medical studies on the relationship between breast cancer and the thyroid gland are increasing in recent years, and the specific conclusions obtained are inconsistent [8, 9]. Therefore, this study intended to explore the clinicopathologic features and risk factors of patients with multiple primary breast cancers and thyroid disease, by mainly analyzing the clinical data of 80 breast cancer patients who were admitted to our hospital from January 2020 to January 2022. The reports are as follows.

## 2. Data and Methods

### 2.1. General Data

An analytic approach of the reviewing method was adopted to analyze the clinical data of 80 breast cancer patients who were admitted to our hospital from January 2020 to January 2022. They were divided into an observation group (breast cancer with thyroid lesions) and a control group (simple breast cancer) according to whether the clinical data were accompanied with thyroid lesions.

#### 2.1.1. Inclusion Criteria

The inclusion criteria are as follows: (1) primary breast cancer is confirmed through clinical diagnosis and laboratory tests; (2) there is no treatment history of chemotherapy, radiotherapy, endocrine therapy, and thyroid disease treatment; (3) thyroid diseases include nodular goiter, thyroid adenoma, and thyroid cancer; and (4) complete clinical and pathological data are available.

#### 2.1.2. Exclusion Criteria

The exclusion criteria are as follows: (1) patients with nonprimary breast cancer; (2) patients who lack some relevant imaging data such as B ultrasound of the thyroid or CT; and (3) patients with unclear consciousness and who could not cooperate with the study.

#### 2.1.3. Included Case Data

Strict investigation was conducted on the inclusion and exclusion criteria in this study. A total of 80 patients were involved in this study, and all of them were female, aged from 24 to 79, with an average age of 47.11 ± 10.11 years. Among them, there were 40 cases of breast cancer combined with a thyroid lesion, accounting for 50.00%, aged from 25 to 79, with the average age of 50.99 ± 10.11 years; there were 40 patients (50.00%) with simple breast cancer, aged from 24 to 78 years old, with an average age of 46.33 ± 10.11 years.

Analyses of specific conditions of patients with breast cancer combined with thyroid lesion are as follows: (1) at the beginning of admission, 40 patients with breast cancer coexisting with thyroid lesion were found, and 35 cases were benign and 5 cases were malignant according to the results of the B ultrasound and CT examination; (2) during the follow-up period, there were 15 cases of thyroid lesions, and 14 cases were benign and 1 case was malignant according to the results of the B ultrasound and CT examination.

### 2.2. Relevant Definitions and Standards

Nodular lesion changes of thyroid lesions are as follows: it is mainly about the morphology description of the thyroid goiter, which can be divided into two types according to the relevant diagnostic criteria—thyroid nodules with malignant signs and benign thyroid nodules. The malignant risk of the malignant signs of thyroid nodules involved this time is between 5% and 90%: (1) Ultrasound examination shows that the edges are irregular; i.e., there are infiltrations, lobulations, and burrs; microcalcification; invasion of thyroid capsule; interrupted marginal calcification; and aspect ratio > 1. (2) The components in the solid nodules/cystic solid nodules are manifested as hypoechoic or solid partial eccentricity.

*Body weight index*: it is also known as the body mass index, which is an evaluation standard currently used internationally to judge the degree of obesity and the health of adult groups. The body mass index could be divided into four types: low body weight is when the body mass index is less than 18.5 kg/m^2^, normal body weight is when the body mass index is between 18.5 kg/m^2^ and 24.0 kg/m^2^, overweight is when the body mass index is between 24.0 kg/m^2^ and 28.0 kg/m^2^, and obesity is when the body mass index is more than 28.0 kg/m^2^.

*Pathological classification of breast cancer*: it is mainly divided according to the WHO pathological definition of breast cancer and its related classification criteria.

The stages of breast cancer are divided according to the specific tumor size, axillary lymph node metastasis, and presence of distant metastasis.

*Immunohistochemistry and molecular typing of breast cancer*: (1) When estrogen receptor and progesterone receptor were positive, the tumor nucleus staining was greater than or equal to 1%; based on the positive internal reference condition, the tumor nucleus staining less than 1% was considered to be negative; uncertainty is determined if normal epithelial cells are stained but tumor nuclei are not stained in the same specimen, or tumor nucleus staining did not exist after multiple inspections in the same specimen. (2) *Nuclear proliferation antigen*: the cells with brown granules in the nucleus were considered to be positive, and a high expression was when the nuclear proliferation antigen was more than or equal to 14%, and the low expression was when the nuclear proliferation antigen was less than 14%. (3) *Human epidermal growth factor receptor-2*: it belongs to the proto-oncogene located on the long arm of human chromosome 17, which needs to be detected by fluorescence in situ hybridization when the result is uncertain. (4) Molecular typing was classified based on the comprehensive expression of the estrogen receptor, progesterone receptor, and nuclear proliferation antigen.

### 2.3. Statistical Process

SPSS 24.0 software was used for analysis. The measurement data was expressed in the form of $\bar{x}\pm s$, and *t* was used for the test. The count data was expressed in the form of %, and *χ*^2^ was used for the test. Logistic regression analysis was used for the multifactor. *P* < 0.05 indicated that the difference had statistical significance.

## 3. Results

### 3.1. Comparison of Clinical Characteristics between the Two Groups

**Figure 1:**
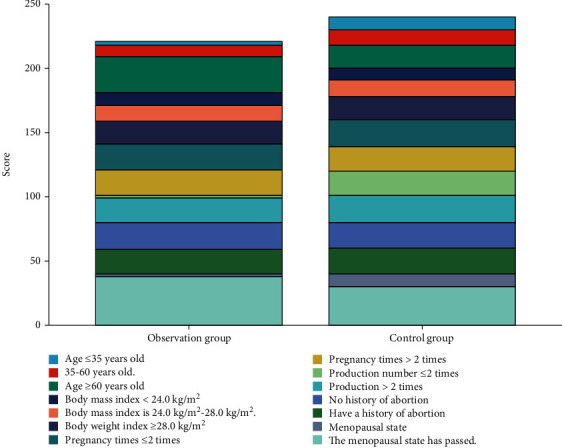
Comparison of clinical features between the two groups.

The number of people aged older than or equal to 60 in the observation group was higher than that in the control group, and there was significant difference in the data of menopausal status between the two groups (*P* < 0.05). There was no statistical difference in the comparison of data, such as body mass index, pregnancy frequency, labor frequency, and abortion history, between the observation group and the control group (*P* > 0.05), as shown in Table 1 and Figure 1.

### 3.2. Comparison of Pathological Types and Stages between the Two Groups

**Figure 2:**
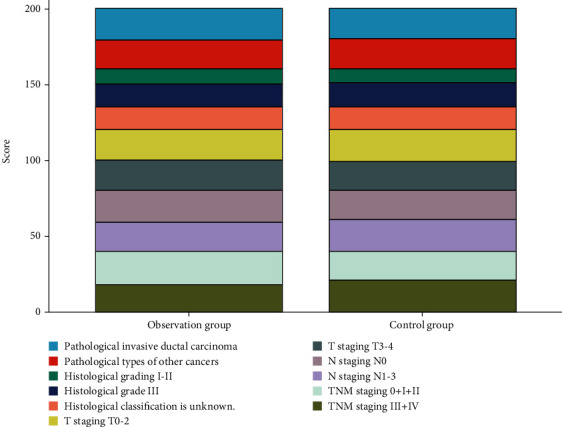
Comparison of pathological types and stages between the two groups.

There was no statistical difference in the data comparison of the pathological type, histological grade, T staging, N staging, and TNM staging between the observation group and the control group (*P* > 0.05), as shown in Table 2 and Figure 2.

### 3.3. Comparison of Molecular Biological Characteristics between the Two Groups

**Figure 3:**
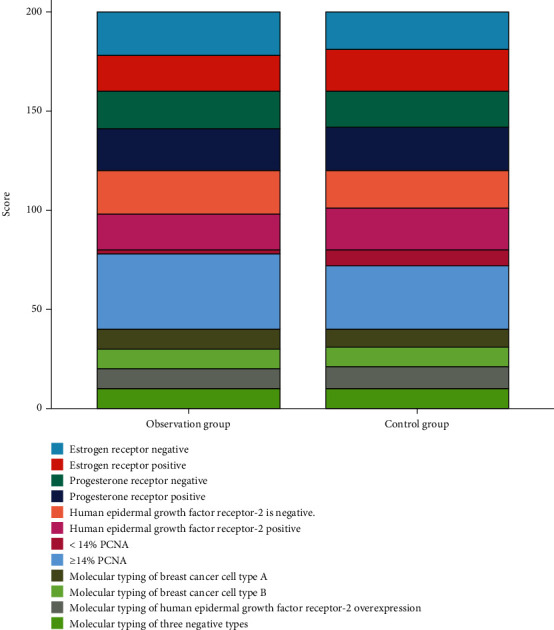
Comparison of molecular biological characteristics between the two groups.

There was statistical difference in the data comparison of the nuclear proliferation antigen between the observation group and the control group (*P* < 0.05). There was no statistical difference in the data comparison of the estrogen receptor, progesterone receptor, human epidermal growth factor receptor-2, and molecular typing between the observation group and the control group (*P* > 0.05), as shown in Table 3 and Figure 3.

### 3.4. Multifactor Analysis

Logistic regression analysis showed that age, menopausal status, and nuclear proliferation antigen index were the high-risk factors for combined thyroid lesions (*P* < 0.05), as shown in Tables 4 and 5.

## 4. Discussion

The concept and research of multiple primary malignant tumors have attracted much attention in the medical field in recent years. It was first proposed in 1989, and the data showed [10] that after the diagnosis and treatment of the patients' first cancer, the chance of developing a second cancer would increase. At present, the specific etiology and pathogenesis of this condition are not very clear, but it is roughly related to many factors, including genetics, environment, and treatment. Breast cancer is a very common kind of female malignant tumor, and it is also the main disease type leading to the death of women, which poses a serious threat to their physical and mental health. With the increasing pace of social life and increasing physical and mental pressure on women, a variety of internal and external factors will affect the endocrine function of the body, and the incidence of endocrine-related thyroid diseases also increases [11]. Both the mammary gland and thyroid gland are closely related to the body hormone levels, while the pituitary gland, ovary, adrenal cortex, etc., which secrete hormones, play an important role in the pathogenesis of breast cancer. Among them, estrogen and progesterone are the most important endocrine hormones that have been proven to influence the pathogenesis of breast cancer based on current research [12, 13]. The thyroid gland is also one of the organs affected by endocrine glands, just like with breast cancer, which is subject to the influence of the hypothalamus-pituitary-gland axis of the human body and the proprioceptive secretion control system, wherein the hypothalamus secretes a thyroid-stimulating hormone-releasing hormone, the pituitary gland secretes a thyroid-stimulating hormone, and the thyroid gland itself secretes hormones to exert effects on the normal morphology of the thyroid gland and its functional maintenance. Thus, the occurrence of breast cancer and thyroid disease may interact with each other, and it is difficult to separate their associations. In the current clinical research data, many researchers have begun to conduct in-depth research on the relationship between breast cancer and thyroid lesions to develop as much research data as possible in order to show the correlation between the two diseases. However, more research is needed to confirm the mechanism behind breast cancer and the thyroid [14, 15].

According to multiple data [16, 17], thyroid nodule lesions occur in 30.7% of males and 39.9% of females, and the incidence will be significantly increased in the female patient population with breast cancer. Among thyroid lesions, compared with the general population, breast cancer patients are more likely to have thyroid lesions that are malignant, generally about seven times that of the general population, which shows that breast cancer patients have a very high risk of thyroid lesions. In this study, there were a total of 40 patients with breast cancer combined with thyroid lesions, including five patients with thyroid lesions of malignant signs. The conclusion of the study was generally consistent with the conclusion in the previous literature. However, the prevalence rate of thyroid cancer in this study was low, which might be related to a small total number of selections and many other factors. In addition, no puncture biopsy was conducted for patients with malignant signs in this study, so there might be a certain deviation.

According to the data [18, 19], the onset age of breast cancer in Chinese women is mainly between 45 and 55 years old, while, in Western countries, it is mainly around 65 years old. The onset age of breast cancer in China has a tendency to change towards results found in Western countries in recent years. Studies have speculated that in 2030, there may be about 27.0% patients over the age of 65 years old diagnosed with breast cancer in China, which will be higher than in previous years. For the postmenopausal elderly, their ovarian function will decrease, together with the reduction of estrogen and progesterone and the transformation of the normal growth rate of mammary cells, leading to the atrophy of the acinus in the body. In addition, their immune ability will be reduced due to age factors, so they are more likely to develop cancer. Therefore, the elderly are also the high-incidence group of thyroid disease. According to the data [20], compared with the population aged 20–30, the incidence of thyroid lesions in the elderly over 70 years old is four times higher. Thus, breast cancer patients with menopause directly affect the prevalence of thyroid disease. In this study, it was also shown that the differences in the age and menopausal status between the two groups were significant (*P* < 0.05). Besides, multivariate analysis showed that age and menopause were the high-risk factors for breast cancer patients complicated with thyroid lesions; that is, the postmenopausal breast cancer patients aged ≥60 years old have a greater chance of thyroid lesions. Between age and menopausal status factors, the postmenopausal status is more instructive than age because all patients above 60 years old have reached menopausal status, while not all patients who are already postmenopausal have reached 60 years old. On the other hand, the cell proliferation antigen, which is a regulatory factor affecting the cell proliferation and differentiation of the body, is also a nuclear proliferation marker with an extremely high-application value at present, which can effectively reflect the proliferation capacity and invasion of tumor cells, and plays a key role in maintaining cell proliferation. Nowadays, when molecular typing is performed to detect breast cancer, the cell proliferation antigen is also one of the important judgment indicators, because some research data have confirmed that the expression of the cell proliferation antigen is closely related to the pathological differentiation, staging, and axillary lymph metastasis of breast cancer tissue, which can become an important indicator for judging the severity and prognosis of breast cancer patients. In addition, the cell proliferation antigen also has a certain expression in the process of thyroid lesions, especially in thyroid papillary carcinoma, which is closely related to the specific tumor size and thyroglobulin antibody. It is generally believed that the higher the expression level of the cell proliferation antigen is, the higher the recurrence rate of the disease will be, which is an effective biological marker for identifying benign and malignant thyroid lesions. The patients with breast cancer and recurrent thyroid cancer also have the phenomenon of high expression of the cell proliferation antigen. This study also indicates that observation, which shows that the nuclear proliferation antigen index belongs to the risk factor for breast cancer combined with thyroid lesions, which may be related to the degree of thyroid cell proliferation and differentiation affected by the high expression of the nuclear proliferation antigen.

According to the data analysis of this study, among the comparisons of clinical data, the number of people aged ≥60 in the observation group was higher than that in the control group, and there was significant difference between the groups in the menopausal status and nuclear proliferation antigen data (*P* < 0.05). There was no statistical difference in the body mass index, pregnancy frequency, labor frequency, abortion history, pathological type, histological grade, T staging, N staging, TNM staging, estrogen receptor, progesterone receptor, human epidermal growth factor receptor-2, molecular typing, and other data between the observation group and the control group (*P* > 0.05). To sum up, for patients with simple breast cancer, age, menopausal status, and nuclear proliferation antigen index are risk factors for developing combined thyroid lesions. Therefore, clinical attention should be paid to the above factors, and risk screening for the above factors should be conducted in advance in the process of clinical treatment to achieve the purpose of improving the prognosis to the greatest extent. In this study, there are also certain research limitations, such as having no thyroid function inspections for breast cancer patients. Therefore, related inspections should be improved in future research to identify the specific mechanism of occurring thyroid lesions in breast cancer, so as to provide a more accurate theoretical basis for the relevant research on clinicopathologic characteristics and risk factors of patients with multiple primary breast cancers and thyroid disease.

## Figures and Tables

**Table 1:** Comparison of clinical characteristics between the two groups (%).

Index		Observation group (*n* = 40)	Control group (*n* = 40)	*χ* ^2^	*P*
Age	≤35 years old	3 (7.50)	10 (25.00)	6.372	0.041
	35–60 years	9 (22.50)	12 (30.00)		
	≥60 years old	28 (70.00)	18 (45.00)		

Body mass index	<24.0 kg/m^2^	10 (25.00)	9 (22.50)	0.0926	0.954
	24.0 kg/m^2^–28.0 kg/m^2^	12 (30.00)	13 (32.50)		
	≥28.0 kg/m^2^	18 (45.00)	18 (45.00)		

Pregnancy times	≤2 times	20 (50.00)	21 (52.50)	0.050	0.823
	>2 times	20 (50.00)	19 (47.50)		

Production number	≤2 times	21 (52.50)	19 (47.50)	0.200	0.655
	>2 times	19 (47.50)	21 (52.50)		

Abortion history	Without	21 (52.50)	20 (50.00)	0.050	0.823
	Have	19 (47.50)	20 (50.00)		

Menopausal status	No menopause	2 (5.00)	10 (25.00)	6.275	0.012
	Menopause	38 (95.00)	30 (75.00)		

**Table 2:** Comparison of pathological types and stages between the two groups (%).

Index		Observation group (*n* = 40)	Control group (*n* = 40)	*χ* ^2^	*P*
Pathological type	Invasive ductal carcinoma	21 (52.50)	20 (50.00)	0.050	0.823
	Other cancers	19 (47.50)	20 (50.00)		

Histological grade	I–II	10 (25.00)	9 (22.50)	0.085	0.958
	III	15 (37.50)	16 (40.00)		
	Unknown	15 (37.50)	15 (37.50)		

T staging	T0-2	20 (50.00)	21 (52.50)	0.050	0.823
	T3-4	20 (50.00)	19 (47.50)		

N staging	N0	21 (52.50)	19 (47.50)	0.200	0.655
	N1-3	19 (47.50)	21 (52.50)		

TNM staging	0+I+II	22 (55.00)	19 (47.50)	0.450	0.502
	III+IV	18 (45.00)	21 (52.50)		

**Table 3:** Comparison of molecular biological characteristics between the two groups (%).

Index		Observation group (*n* = 40)	Control group (*n* = 40)	*χ* ^2^	*P*
Estrogen receptor	Negative	22 (55.00)	19 (47.50)	0.450	0.502
	Positive	18 (45.00)	21 (52.50)		

Progesterone receptor	Negative	19 (47.50)	18 (45.00)	0.050	0.823
	Positive	21 (52.50)	22 (55.00)		

Human epidermal growth factor receptor-2	Negative	22 (55.00)	19 (47.50)	0.450	0.502
	Positive	18 (45.00)	21 (52.50)		

Nuclear proliferation antigen	<14%	2 (5.00)	8 (20.00)	4.114	0.043
	≥14%	38 (95.00)	32 (80.00)		

Molecular typing	Breast cancer cell type A	10 (25.00)	9 (22.50)	0.100	0.992
	Breast cancer cell type B	10 (25.00)	10 (25.00)		
	Human epidermal growth factor receptor-2 overexpression	10 (25.00)	11 (27.50)		
	Three negative types	10 (25.00)	10 (25.00)		

**Table 4:** Variable assignment of influencing factors for multiple primary breast cancers complicated with thyroid lesions.

Variable	Assignment
Dependent variable	
Breast cancer	Simple breast cancer = 0; combined thyroid lesions = 1
Independent variable	
Age	≤35 years old, ≥60 years old = 0; 35–60 years = 1
Menopausal status	No menopause = 0; menopause = 1
Nuclear proliferation antigen index	Positive = 0; negative = 1

**Table 5:** Multifactor analysis.

Correlative factor	*β*	Standard deviation	Wald	*P*	OR	95% CI
Age	1.615	0.597	7.318	0.011	3.217	2.021–6.119
Menopausal status	1.446	0.611	5.601	0.012	3.187	2.077–4.177
Nuclear proliferation antigen index	1.721	0.356	23.370	0.003	2.995	1.336–4.369

## Data Availability

The labeled dataset used to support the findings of this study are available from the corresponding author upon request.
